# Body mass index and waist circumference in relation to risk of recurrence and progression after non‐muscle invasive bladder cancer

**DOI:** 10.1002/cam4.6620

**Published:** 2023-10-06

**Authors:** Moniek van Zutphen, Ivy Beeren, Katja K. H. Aben, Antoine G. van der Heijden, J. Alfred Witjes, Lambertus A. L. M. Kiemeney, Alina Vrieling

**Affiliations:** ^1^ Department for Health Evidence Radboud University Medical Center Nijmegen The Netherlands; ^2^ Netherlands Comprehensive Cancer Organisation Utrecht The Netherlands; ^3^ Department of Urology Radboud University Medical Center Nijmegen The Netherlands

**Keywords:** abdominal, non‐muscle invasive bladder cancer, obesity, progression, recurrence

## Abstract

**Background:**

Obesity may be associated with increased risk of recurrence and progression in patients with non‐muscle invasive bladder cancer (NMIBC), but evidence is limited and inconsistent. We examined the associations of body mass index (BMI), waist circumference, and waist‐to‐hip ratio (WHR) with risk of recurrence and progression among patients with NMIBC.

**Methods:**

This prospective study included 1029 patients diagnosed with primary NMIBC between 2014 and 2017. Patients reported weight 2 years before diagnosis at baseline, and weight, waist and hip circumference at 3 months postdiagnosis. Associations were quantified using Cox proportional hazard analyses, adjusted for clinical and lifestyle characteristics.

**Results:**

More than half of patients were overweight (49%) or obese (19%) after diagnosis. During a median follow‐up time of 3.6 years, 371 patients developed ≥1 recurrence and 53 experienced progression. No associations with recurrence were observed for BMI (HR_per 5 kg/m2_ 0.94; 95% CI 0.82, 1.07), waist circumference (HR_per 10 cm_ 0.95; 95% CI 0.86, 1.05), or WHR (HR_per 0.1 unit_ 0.90; 95% CI 0.76, 1.06). In contrast, higher BMI was associated with a 40% increased risk of progression, with only the 2‐year prediagnosis association reaching statistical significance (HR_per 5 kg/m2_ 1.42; 95% CI 1.09, 1.84). No associations for pre‐to‐postdiagnosis weight change were found.

**Conclusion:**

General and abdominal obesity were not associated with recurrence risk among patients with NMIBC, but might be associated with increased risk of progression. Studies with sufficient sample size to stratify by tumor stage and treatment are needed to better understand whether and how obesity could influence prognosis.

## 1 INTRODUCTION

With 570,000 new cases each year, bladder cancer is the 10th most commonly diagnosed cancer worldwide and the 6th most common type of cancer in men.^1^ Nearly 75% of diagnosed bladder cancer cases are non‐muscle invasive.^2^ These patients have a good survival, but are at high risk of tumor recurrence.^3^ This necessitates a burdensome treatment and follow‐up program which causes non‐muscle invasive bladder cancer (NMIBC) to be one of the most expensive cancers in terms of lifetime treatment costs.^4^ For bladder cancer, acknowledged risk factors for worse oncologic outcomes are primary tumor characteristics, including higher tumor stage, higher grade, multiplicity, larger size, and concomitant carcinoma in situ.^5^ Lifestyle factors such as smoking and obesity may also be relevant for the prognosis of NMIBC and may contribute to more personalized follow‐up schemes or interventions.^6^

There is limited and inconsistent evidence regarding the association between obesity and risk of NMIBC recurrence and progression. Seven cohort studies showed that higher body mass index (BMI) was associated with higher risk of recurrence,^7^, ^8^, ^9^, ^10^, ^11^, ^12^, ^13^ of which five studies showed statistically significant associations.^7^, ^8^, ^9^, ^10^, ^11^ In contrast, two studies reported no associations.^14^, ^15^ A meta‐analysis of five cohort studies concluded that obesity was associated with a 2‐fold increased risk of recurrence and an 88% increased risk of progression compared to healthy weight patients with NMIBC.^16^ However, all studies only assessed BMI around time of diagnosis and most studies did not adjust for smoking status or other lifestyle factors, which might lead to biased outcomes.

To date, studies have not focused on other measures of obesity than BMI and have not included changes in weight over time. Using data of a prospective cohort study, we examined the associations between several measures of obesity (pre‐and postdiagnosis BMI, postdiagnosis waist circumference and waist‐to‐hip ratio [WHR]) with the risk of recurrence and progression in patients with NMIBC, while adjusting for smoking and other lifestyle factors. Furthermore, we examined whether pre‐to‐post diagnosis weight change was associated with NMIBC recurrence and progression.

## 2 METHODS

### 2.1 Study design and population

**FIGURE 1:**
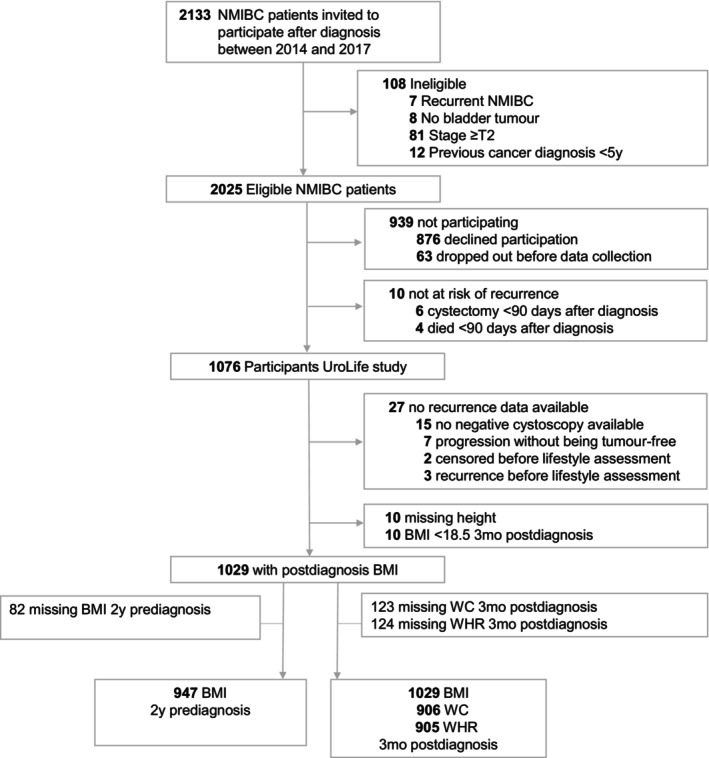
Flowchart representing patient selection for the current study. Anthropometry data was available at two different time‐points: 2‐years prediagnosis and 3‐months postdiagnosis. UroLife, Urothelial cell cancer; Lifestyle, prognosis and quality of life; NMIBC, non‐muscle invasive bladder cancer; BMI, body mass index; WC, waist circumference; WHR, waist‐to‐hip ratio.

We used data from the UroLife study, a prospective multicenter cohort study among patients diagnosed with primary NMIBC.^17^ Patients were recruited in 22 hospitals in the Netherlands between May 2014 and April 2017. Eligible patients were identified through the Netherlands Cancer Registry hosted by the Netherlands Comprehensive Cancer Organization using notification lists of the Pathological Anatomical National Automated Archive (PALGA Foundation). Patients were eligible if they were between 18 and 80 years old, Dutch speaking, diagnosed with a histologically confirmed primary stage Ta, T1, or Tis urothelial carcinoma of the tumor, and underwent a transurethral resection of the bladder tumor (TURBT). Exclusion criteria are shown in Figure 1. Approximately 4 weeks after diagnosis, patients were invited to participate in the UroLife study. Patients who agreed to participate provided written informed consent. Ethical approval was provided by the Committee for Human Research region Arnhem‐Nijmegen (CMO 2013–494).

### 2.2 Assessment of anthropometrics

Information on anthropometrics was collected at 2 time points via self‐reported web‐based or paper‐and‐pencil based questionnaires. First, at approximately 6 weeks after diagnosis (baseline), information on current height (and body weight) and body weight 2 years before diagnosis was collected. Second, at 3 months after diagnosis information on current body weight, waist circumference, and hip circumference was collected. Waist (midway between the lowest rib and the iliac crest) and hip (widest part) circumference were measured in centimeters with a tape sent to participants. Anthropometric measures were used as continuous and predefined categorical variables, that is, obesity (BMI ≥30.0), overweight (BMI ≥25‐ < 30), and healthy weight (BMI ≥18.5– < 25 kg/m^2^). Patients with BMI <18.5 kg/m^2^ (*n* = 10) were excluded from the analyses (Figure 1), as this underweight group was too small for performing statistical analyses. If BMI at 3 months after diagnosis was missing (*n* = 119), we used BMI assessed at baseline (correlation between both measures of BMI was 0.99). Pre‐to‐postdiagnosis weight change was calculated as the change in weight between 2 years prediagnosis and 3 months postdiagnosis and expressed as percentage weight change. Weight change was defined as weight loss ≥5%, weight stable −5% to 5%, and weight gain ≥5%, as ≥5% weight loss has been proven to have significant health benefits and is generally accepted as criterion for clinically meaningful weight change.^18^ Waist circumference was categorized into three groups based on sex‐specific cutoff points commonly used for cancer prevention recommendations,^19^ while WHR was categorized into three groups based on sex‐specific tertiles. Waist circumference and WHR are measures of abdominal obesity, while BMI is a measure of general obesity.

### 2.3 Outcome assessment

Information on recurrences and progression was collected from medical records by trained data managers from the Netherlands Cancer Registry in February–March 2021. A recurrence was defined as the first new bladder tumor after being tumor‐free. Being tumor‐free was defined by the date of radical TURBT (either primary TURBT or re‐TURBT) in case of Ta and T1 tumors and by the date of the first tumor‐negative cystoscopy after primary TURBT in case of (concomitant) carcinoma in situ (CIS). Progression was defined as the first occurrence of stage or grade progression.^20^ Patients were censored at (a) last contact between patient and urologist, (b) radical cystectomy in absence of recurrence/progression, or (c) diagnosis of another type of cancer with metastasis, whichever came first.

### 2.4 Covariate assessment

At baseline, patients reported sociodemographic information and whether they were ever diagnosed with diabetes mellitus. Smoking status was reported at baseline (reflecting pre‐diagnosis smoking) and 3 months postdiagnosis. Physical activity was assessed using the validated SQUASH questionnaire.^21^, ^22^, ^23^, ^24^, ^25^, ^26^, ^27^ The reference period was a normal week in the months before diagnosis (at baseline) or the previous 3 months (during follow‐up). Total minutes per week of moderate‐to‐vigorous physical activity were calculated based on leisure‐time (cycling, gardening, odd jobs, and sports with a metabolic equivalent value ≥3) and commuting (walking and cycling) activities. Dietary intake was assessed using a 163‐item validated semiquantitative food frequency questionnaire (FFQ) developed by Wageningen University.^28^, ^29^, ^30^ The reference period for the FFQ was the year before diagnosis (at baseline) or the previous month (during follow‐up). In the baseline FFQ, fruit and vegetable intake were queried separately for summer and winter. To limit seasonal variability between baseline and follow‐up, we calculated baseline fruit and vegetable intake based on reported intake in the season that matched the season of follow‐up assessment. To assess fruit and vegetable intake, alcoholic drinks, and total energy intake, we combined frequencies of intake with standard portion sizes and household measures.^28^ Intakes of alcohol and total energy were calculated based on the 2011 Dutch Food Composition Database.^31^

Clinical data, such as tumor stage, differentiation grade, presence of (concomitant) CIS, tumor focality, and treatment, was collected from medical records by trained data managers of the Netherlands Cancer Registry. Participants were divided into low‐, intermediate‐ and high‐risk groups according to the European Association of Urology (EAU) guidelines 2019 based on stage, grade, CIS, and focality,^5^ without considering tumor size (not available) and the recurrent nature of the tumor (only primary tumors included).

### 2.5 Statistical analyses

First recurrence was the primary endpoint; progression was a secondary endpoint. Cox proportional hazard regression models were used to calculate hazard ratios (HRs) and 95% confidence intervals (CIs) for the associations of anthropometry measures with recurrence and progression. Follow‐up time began at baseline or follow‐up questionnaire completion (progression) or at the first day of being tumor‐free in case a person was not tumor‐free at questionnaire completion (recurrence). In addition, we analyzed multiple recurrences with an extended Cox model for recurrent event data (gap time—unrestricted (GT‐UR) model with a common baseline hazard and random effect).^32^, ^33^ As the recurrence‐specific baseline hazards were similar for all recurrence numbers, we selected a model with a common baseline hazard; analysis time was reset at each recurrence. The GT‐UR model has a slightly different HR interpretation compared with the well‐known Cox model; HRs are the increase or decrease of recurrence risk since the last event (either primary tumor or previous recurrence). The full models used to evaluate BMI 2 years prediagnosis were adjusted for age, sex, education level (low, intermediate, high), diabetes at diagnosis, EAU risk group (low, intermediate, high), initial treatment (only TURBT, only TURBT + single instillation, chemotherapy, BCG), baseline smoking status (never, former, current), moderate‐to‐vigorous physical activity, and fruit and vegetable, alcohol and energy intake. In the full models of postdiagnosis anthropometry (BMI, waist circumference, or WHR), we included the same covariates as in the analyses of prediagnosis BMI, except that smoking status, dietary intake, and physical activity were obtained from the postdiagnosis questionnaire. Weight change models were adjusted for the same covariates as the postdiagnosis models, with the addition of 2 years prediagnosis BMI. Besides diabetes, we did not consider any other components of the metabolic syndrome (like hypertension or hyperlipidemia) as these are not generally considered as risk factors for cancer recurrence and progression. To test for linear trends, the median score of each category was assigned to all participants within that category and entered as a continuous exposure in the Cox models. The proportional hazards assumption was tested using Schoenfeld's global test, and no statistically significant violations were detected. Multiple imputation (MICE) with 20 iterations, including Nelson‐Aalen estimators and survival outcomes, was used to deal with missing self‐reported data (exposures and covariates) in the Cox models.^34^

Subgroup analyses were performed to explore whether the associations between BMI at 3 months postdiagnosis and recurrence were modified by age at diagnosis, sex, smoking status, diabetes, EAU risk group, tumor stage, tumor grade, and initial treatment.

All analyses were performed in R for Windows version 4.1.3 (i.e., packages “MICE”, “survival”, “coxme”). *p*‐values ≤0.05 were considered statistically significant.

## 3 RESULTS

Of the 2133 invited patients, 2025 patients were eligible and 1076 (53%) agreed to participate (Figure 1). A total of 1029 participants were eligible for the analyses of the current study.

Baseline characteristics for the total study population and according to BMI 3 months postdiagnosis are listed in Table 1. The median age at NMIBC diagnosis was 67 years and 80% was male. Most participants had stage Ta disease (76%). The median BMI 3 months postdiagnosis was 26.6 kg/m^2^, with 49% being overweight and 19% being obese. Tumor characteristics did not differ by BMI classes. Patients with obesity were more likely to have diabetes while they were less likely to have a high educational level and to have never smoked, and they spent less time in moderate‐to‐vigorous physically activity compared with patients with a healthy weight. Participants included in the current analysis did not differ from eligible and invited non‐participants with respect to age, sex, tumor stage, and tumor grade (data not shown). Pearson correlation coefficients of BMI 3 months postdiagnosis with waist circumference, WHR, and BMI 2‐year prediagnosis were 0.79, 0.38, and 0.89, respectively.

**TABLE 1:** Population characteristics of non‐muscle invasive bladder cancer patients at 3‐months postdiagnosis overall and by BMI category (*n* = 1029).

	BMI 3‐months postdiagnosis (kg/m^2^)			
	Total	18.5‐25	25–29.9	≥30
*N*	1029	332 (32%)	500 (49%)	197 (19%)
Age at diagnosis, y	67 (61, 72)	68 (60, 73)	67 (61, 72)	68 (62, 72)
Men	827 (80%)	249 (75%)	428 (86%)	150 (76%)
Education				
Low	506 (49%)	151 (45%)	248 (50%)	107 (54%)
Intermediate	274 (27%)	83 (25%)	136 (27%)	55 (28%)
High	249 (24%)	98 (30%)	116 (23%)	35 (18%)
Stage				
Tis	24 (2%)	8 (2%)	12 (2%)	4 (2%)
Ta	785 (76%)	261 (79%)	374 (75%)	150 (76%)
T1	220 (21%)	63 (19%)	114 (23%)	43 (22%)
Grade^1^				
Low	662 (65%)	228 (69%)	301 (60%)	133 (68%)
High	364 (35%)	102 (31%)	198 (40%)	64 (32%)
Concomitant CIS	98 (10%)	27 (8%)	59 (12%)	12 (6%)
Multifocal tumor^2^	298 (29%)	92 (28%)	148 (30%)	58 (29%)
EAU risk group				
Low	186 (18%)	68 (20%)	79 (16%)	39 (20%)
Intermediate	433 (42%)	147 (44%)	202 (40%)	84 (43%)
High	410 (40%)	117 (35%)	219 (44%)	74 (38%)
Initial treatment				
Only TURBT	193 (19%)	70 (21%)	85 (17%)	38 (19%)
TURBT + single instillation^3^	334 (32%)	115 (35%)	150 (30%)	69 (35%)
TURBT + chemotherapy instillations	227 (22%)	72 (22%)	107 (21%)	48 (24%)
TURBT + BCG	275 (27%)	75 (23%)	158 (32%)	42 (21%)
Diabetes at diagnosis^4^	124 (12%)	24 (7%)	50 (10%)	50 (26%)
Smoking^5^				
Never	165 (18%)	70 (23%)	73 (17%)	22 (12%)
Former	602 (66%)	175 (58%)	301 (70%)	126 (70%)
Current	146 (16%)	58 (19%)	57 (13%)	31 (17%)
BMI 2y prediagnosis^6^, kg/m^2^	26.6 (24.5, 29.1)	23.7 (22.1, 24.7)	27.1 (26.1, 28.7)	32.1 (30.7, 34.3)
BMI 3mo postdiagnosis, kg/m^2^	26.6 (24.4, 29.1)	23.5 (22.0, 24.3)	27.1 (26.0, 28.3)	32.0 (30.9, 34.4)
Waist circumference^7^, cm	99 (92, 107)	90 (86, 95)	100 (95, 106)	113 (106, 122)
Waist‐to‐hip ratio^8^	0.98 (0.93, 1.02)	0.95 (0.90, 0.99)	0.99 (0.95, 1.03)	1.02 (0.97, 1.07)
Physical activity^9^, min/wk	450 (180, 900)	503 (180, 960)	480 (210, 900)	320 (120, 678)
Fruits and vegetables^10^, g/d	206 (130, 292)	205 (136, 307)	205 (126, 293)	212 (136, 285)
Alcohol drinker^10^, %	80%	80%	83%	74%
Alcohol^11^, g/d	14 (7, 27)	13 (6, 26)	15 (4, 27)	16 (5, 30)
Total energy intake^10^, kcal/d	2057 (1683, 2479)	2114 (1683, 2543)	2042 (1703, 2472)	2046 (1610, 2402)

Abbreviations: BCG, bacillus calmette guérin; BMI, body mass index; CIS, carcinoma in situ; cm, centimeter; d, day; EAU, European Association of Urology; g, grams; kcal, kilocalorie; kg, kilograms; m, meter; min, minutes; mo, month; TURBT, transurethral resection of the bladder tumor; WCRF/AICR, World Cancer Research Fund/American Institute of Cancer Research; wk, week; y, year. Values are medians (IQRs) or n (%).

^1^ Data of 3 participants were missing/unknown.

^2^ Data of 7 participants were missing/unknown.

^3^ Single chemotherapy instillation within 1 day after TURBT.

^4^ Data of 11 participants were missing/unknown.

^5^ Data of 116 participants were missing/unknown.

^6^ Data of 82 participants were missing/unknown.

^7^ Data of 123 participants were missing/unknown.

^8^ Data of 124 participants were missing/unknown.

^9^ Data of 126 participants were missing/unknown.

^10^ Data of 130 participants were missing/unknown.

^11^ Among drinkers only.

During a median (IQR) follow‐up time of 3.6 (1.5–4.4) years, 371 patients (36%) experienced at least one tumor recurrence. Only 53 patients (5%) experienced progression during a median (IQR) follow‐up time of 4.1 (3.6–4.8) years.

### 3.1 General obesity and recurrence

**FIGURE 2:**
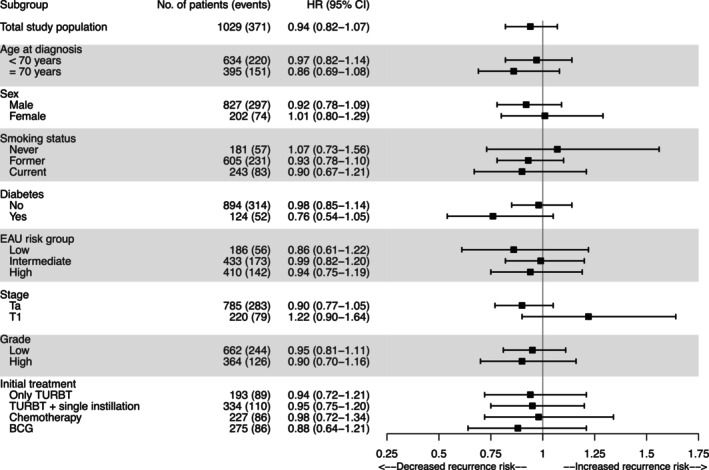
Multivariable HRs and 95% CIs of first recurrence per 5 kg/m^2^ increase in BMI at 3 months after diagnosis, for the total study population and stratified by age at diagnosis, sex, smoking status, diabetes, EAU risk group, tumor stage, tumor grade, and initial treatment. Models were adjusted for age at diagnosis, sex, education, postdiagnosis smoking status, diabetes at diagnosis, postdiagnosis energy intake, EAU risk group (based on stage, grade, CIS, multifocality), and initial treatment (only TURBT, TURBT + single instillation, chemotherapy, BCG), except in models stratified by these variables.

BMI was not associated with risk of recurrence (Table 2). The adjusted HRs (model 3) for overweight and obesity at 3 months postdiagnosis, with healthy weight as reference, were 0.98 (95% CI: 0.77, 1.24) and 0.89 (95% CI: 0.65, 1.21), respectively. The analysis for continuous BMI at 3 months postdiagnosis yielded a HR of 0.94 (95% CI: 0.82, 1.07) per 5 kg/m^2^ increase in BMI. Results were similar for BMI 2 years prediagnosis (HR _per 5 kg/m2_ 0.93; 95% CI 0.82, 1.06). There was little evidence of confounding by clinical and treatment characteristics, smoking status, and other lifestyle factors compared with the model only accounting for age at diagnosis and sex (Table 2, model 1). Furthermore, results did not meaningfully differ when analyzing multiple recurrences instead of first recurrences (Table S1). Subgroup analyses showed no statistically significant effect modification of the associations between BMI 3 months postdiagnosis and recurrence by age, sex, smoking status, level of education, diabetes at diagnosis, EAU risk group, or treatment (Figure 2). However, higher BMI might be associated with an increased recurrence risk among the subgroup of patients with stage T1 NMIBC.

**TABLE 2:** Hazard ratios (HR) for the association of obesity with risk of first bladder cancer recurrence.

Measure	N	Events/person‐years	Model 1 HR (95% CI)	Model 2 HR (95% CI)	Model 3 HR (95% CI)
BMI 2 years prediagnosis, kg/m^2^					
18.5 to <25.0	317	120/974	1.00 (ref)	1.00 (ref)	1.00 (ref)
25.0 to <30.0	501	180/1560	0.93 (0.74, 1.18)	0.95 (0.75, 1.21)	0.96 (0.76, 1.22)
≥30	210	70/665	0.84 (0.62, 1.13)	0.79 (0.58, 1.08)	0.82 (0.60, 1.13)
P‐trend			0.25	0.14	0.23
Per 5 kg/m^2^	1029	371/3199	0.93 (0.83, 1.06)	0.92 (0.81, 1.04)	0.93 (0.82, 1.06)
BMI 3 month postdiagnosis, kg/m^2^					
18.5 to <25.0	332	120/1012	1.00 (ref)	1.00 (ref)	1.00 (ref)
25.0 to <30.0	500	181/1572	0.97 (0.77, 1.23)	0.99 (0.79, 1.26)	0.98 (0.77, 1.24)
≥30	197	70/615	0.95 (0.71, 1.28)	0.90 (0.66, 1.23)	0.89 (0.65, 1.21)
P‐trend			0.74	0.53	0.46
Per 5 kg/m^2^	1029	371/3199	0.96 (0.85, 1.09)	0.95 (0.83, 1.08)	0.94 (0.82, 1.07)
Waist circumference 3 month postdiagnosis, cm					
<94 M and < 80 F	227	81/696	1.00 (ref)	1.00 (ref)	1.00 (ref)
94 to <102 M and 80 to <88 F	289	109/907	1.03 (0.76, 1.38)	1.06 (0.79, 1.43)	1.04 (0.77, 1.41)
≥102 M and ≥ 88 F	513	181/1596	0.95 (0.72, 1.25)	0.94 (0.71, 1.24)	0.90 (0.68, 1.20)
P‐trend			0.60	0.50	0.33
Per 10 cm	1029	371/3199	0.97 (0.89, 1.06)	0.96 (0.87, 1.05)	0.95 (0.86, 1.05)
Waist‐to‐hip ratio 3 month postdiagnosis					
Sex‐specific tertile 1^a^	333	126/1046	1.00 (ref)	1.00 (ref)	1.00 (ref)
Sex‐specific tertile 2^b^	343	130/1015	1.07 (0.83, 1.38)	1.09 (0.84, 1.42)	1.07 (0.82, 1.39)
Sex‐specific tertile 3^c^	353	115/1138	0.86 (0.66, 1.13)	0.83 (0.63, 1.10)	0.82 (0.62, 1.08)
P‐trend			0.21	0.13	0.10
Per 0.1 unit	1029	371/3199	0.92 (0.78, 1.08)	0.91 (0.77, 1.07)	0.90 (0.76, 1.06)

*Note*: Model 1: adjusted for age at diagnosis and sex. Multiple imputation with 20 iterations was used for missing covariates and/or BMI 2y prediagnosis, waist circumference, and waist‐to‐hip ratio.

*Note*: Model 2: Model 1 + further adjustment for education, smoking status, diabetes at diagnosis, EAU risk category (based on stage, grade, CIS, multifocality), and initial treatment (only TURBT, single instillation, chemotherapy, BCG).

*Note*: Model 3: Model 2 + further adjustment for energy intake, fruit & vegetable intake, alcohol intake, and physical activity.

^a^ Cutoffs: <0.967 M (male) and <0.861 F (female).

^b^ Cutoffs: 0.967 to 1.019 M and 0.861 to 0.926 F.

^c^ Cutoffs: >1.019 M and >0.926 F.

### 3.2 Abdominal obesity and recurrence

Waist circumference and WHR at 3 months postdiagnosis were not associated with risk of recurrence (Table 2). The analysis for continuous waist circumference yielded a HR of 0.95 (95% CI: 0.86, 1.05) per 10 cm increase and for WHR a HR of 0.90 (95% CI: 0.76, 1.06) per 0.1‐unit increase. Results did not differ meaningfully when we analyzed multiple recurrences instead of first recurrences (Table S1).

### 3.3 Obesity and progression

Higher BMI was associated with a 40% increased risk of progression, although only the 2 years prediagnosis association reached statistical significance (HR _per 5 kg/m2_ 1.42; 95% CI 1.09, 1.84) (Table 3). The HR for waist circumference was 1.12 (95% CI: 0.87, 1.44) per 10 cm increase and for WHR 1.29 (95% CI: 0.84, 1.97) per 0.1‐unit increase.

**TABLE 3:** Hazard ratios (HR) for the association of obesity with risk of bladder cancer progression.

	N	Events / person‐years	Model 1 HR (95% CI)	Model 2 HR (95% CI)
BMI, per 5 kg/m^2^				
2 years prediagnosis	1029	53/4095	1.30 (1.02, 1.65)	1.42 (1.09, 1.84)
3 month postdiagnosis	1029	53/4095	1.26 (0.93, 1.71)	1.37 (0.99, 1.89)
Waist circumference, per 10 cm				
3 month postdiagnosis	1029	53/4095	1.09 (0.86, 1.38)	1.12 (0.87, 1.44)
Waist‐to‐hip ratio, per 0.1 unit				
3 month postdiagnosis	1029	53/4095	1.29 (0.86, 1.94)	1.29 (0.84, 1.97)

*Note*: Model 1: adjusted for age at diagnosis and sex and using multiple imputation with 20 iterations for missing covariates and/or BMI 2y prediagnosis, waist circumference, waist‐to‐hip ratio.

*Note*: Model 2: Model 1 + further adjustment for education, smoking status, diabetes at diagnosis, EAU risk category (based on stage, grade, CIS, multifocality), and initial treatment (only TURBT, single instillation, chemotherapy, BCG).

### 3.4 Pre‐to‐postdiagnosis weight change

Weight change from 2 years prediagnosis to 3 months postdiagnosis was neither associated with risk of recurrence (HR _per 5%_ 1.02; 95% CI: 0.93, 1.13) nor progression (HR _per 5%_ 0.88; 95% CI 0.69, 1.13) (Table 4). [Correction added on November 16, 2023 after first online publication. The values in the previous sentence has been updated in this version.] However, it should be noted that only 28% of participants experienced a weight change ≥5%.

**TABLE 4:** Hazard ratios (HR) for the association of pre‐to‐post diagnosis weight change with risk of bladder cancer recurrence and progression.

	N	Events/person‐years	Model 1 HR (95% CI)	Model 2 HR (95% CI)	Model 3 HR (95% CI)
First recurrence					
Weight loss (≥5%)	162	53/527	0.87 (0.64, 1.19)	0.81 (0.59, 1.12)	0.87 (0.63, 1.22)
Weight stable (−5% to 5%)	746	276/2283	1.00 (ref)	1.00 (ref)	1.00 (ref)
Weight gain (≥5%)	121	42/390	0.97 (0.68, 1.37)	0.92 (0.65, 1.31)	0.90 (0.63, 1.29)
P‐trend			0.58	0.49	0.84
Per 5% weight gain	1029	371/3199	1.04 (0.95, 1.14)	1.05 (0.95, 1.15)	1.02 (0.93, 1.13)
Progression					
Per 5% weight gain	1029	53/4095	0.90 (0.72, 1.12)	0.88 (0.69, 1.13)	–

*Note*: Model 1: adjusted for age at diagnosis and sex and using multiple imputation with 20 iterations for missing covariates and/or weight change.

*Note*: Model 2: Model 1 + further adjustment for education, smoking status, diabetes at diagnosis, EAU risk category (based on stage, grade, CIS, multifocality), and initial treatment (only TURBT, single instillation, chemotherapy, BCG).

*Note*: Model 3: Model 2 + further adjustment for energy intake, fruit & vegetable intake, alcohol intake, physical activity, and BMI 2y prediagnosis.

## 4 DISCUSSION

In this prospective study among 1029 patients diagnosed with primary NMIBC, BMI, waist circumference, and WHR were not associated with risk of recurrence. In contrast, higher BMI tended to be associated with an increased risk of progression. Weight change from 2‐years prediagnosis to 3 months postdiagnosis was neither associated with recurrence nor progression.

### 4.1 In relation to current literature

In contrast to our results, seven out of nine previous observational studies in patients with NMIBC showed that higher BMI was associated with an increased risk of recurrence.^7^, ^8^, ^9^, ^10^, ^11^, ^12^, ^13^ Similar to our results, two studies found no associations.^14^, ^15^ Three studies reported that obesity was associated with a 2.5‐ to 5‐fold increased risk of recurrence compared to healthy weight patients.^7^, ^8^, ^9^ Three other studies reported a 1.2‐ to 1.8‐fold increased risk of recurrence.^10^, ^11^, ^12^ These studies slightly differed by the included study populations. The three studies reporting the strongest associations only included patients with T1 high grade disease and/or those treated with maintenance Bacillus Calmette‐Guérin (BCG) immunotherapy.^7^, ^8^, ^9^ The other three studies included patients with tumor stage Ta, T1, and Tis independent of disease grade and treatment.^10^, ^11^, ^12^ Our subgroup analyses among patients with stage T1 indicated that higher BMI was associated with a statistically nonsignificant increased recurrence risk, while this association was not observed among patients with stage Ta disease. No association for patients treated with BCG was found. However, power for these subgroup analyses was limited. From the previous studies reporting a statistically significant increased risk of recurrence with obesity, only one was adjusted for smoking status.^11^ Two other studies taking smoking into account reported either a statistically nonsignificant increased risk (HR 1.22; 95% CI 0.80, 1.87)^12^ or no association (HR 1.02; 95% CI 0.76, 1.38)^14^ with obesity. Our risk estimates did neither meaningfully change when smoking status was excluded from our model, nor by exclusion of other lifestyle factors (diet and physical activity). Variation in upper BMI range is not expected to explain differences in observed associations between studies. Studies showing that obesity was associated with increased risk of recurrence not only included study populations from the United States,^7^, ^8^ but also from Europe,^9^ Turkey,^11^ and China^10^ where BMIs >35 are less common than in the United States. Furthermore, similar, but slightly lower, risk estimates were reported for overweight patients.^9^, ^10^, ^12^, ^13^ Studies with sufficient sample size to stratify by tumor stage and treatment are needed to explain heterogeneity between study results.

In line with our results, a meta‐analysis of three cohort studies showed that obesity, compared to healthy weight, was associated with an increased risk of progression in patients with NMIBC.^16^ In contrast, two other studies reported either no association^14^ or a protective association.^15^ We had limited power to study risk of NMIBC progression, as we observed relatively few events (n = 53). Therefore, anthropometrics were only analyzed as continuous variables (and not as categorical variables). Within our study, only the association between BMI 2‐years prediagnosis and recurrence reached statistical significance. We cannot rule out that this positive association is due to chance. The recruitment of additional stage T1 patients and extension of follow‐up time for our cohort will increase the power of future progression analyses.

This was the first study that examined the association between weight change and NMIBC prognosis. We found that weight change from 2 years prediagnosis to 3 months postdiagnosis was neither associated with risk of recurrence nor with progression. However, it should be noted that only 28% of participants experienced a weight change ≥5%. For cancer patients, it is important to know whether changing one's lifestyle after diagnosis can lower the risk of recurrence and progression. Additional studies are needed to further examine whether obesity after NMIBC diagnosis and changes therein can impact prognosis.

### 4.2 Weaknesses and strengths of the study

Our study had several limitations. First, anthropometrics were self‐reported at each time point. Generally, errors are present in self‐reported anthropometric data; some people underreport, while others overreport and the magnitude of measurement error in waist circumference is unclear.^35^ However, since anthropometric data were reported before the occurrence of an event, we do not expect these errors to be different between patients with or without an event. Second, although body weight was assessed at several timepoints, obesity at these timepoints might not reflect obesity later during the NMIBC trajectory. However, our group previously concluded that BMI did not significantly change between 3 and 15 months after NMIBC diagnosis.^36^ Third, we did not perform competing risk analyses, although death could prevent a recurrence diagnosis. As only 70 people died without recurrence within 5 years after diagnosis, it seems unlikely that our observed associations would be affected by the competing risk of death. Fourth, although the study was among the largest compared to other cohort studies on the same topic, power was still limited for subgroup analyses and for the outcome progression. Finally, as with all observational studies, we cannot eliminate the possibility of residual confounding even though we were able to include more possible confounders as most other studies on this topic.

Strengths of our study compared with previous studies are the inclusion of multiple measures of obesity and adjustment of our analyses for smoking status and other lifestyle factors. In contrast to previous studies, our study assessed the effects of both general and abdominal obesity. We observed similar associations with NMIBC outcomes for both general and abdominal obesity. Abdominal obesity, measured by waist circumference or WHR, is associated with adverse metabolic profiles such as insulin resistance and systemic inflammation, which are both linked to tumor growth.^37^ Furthermore, relative visceral fat area and degree of systemic inflammation may impact response to BCG treatment.^38^ However, mechanisms underlying the association of abdominal obesity with cancer outcomes may differ by cancer type, and the exact mechanisms for NMIBC recurrence and progression remain to be elucidated. In contrast to most previous studies, we adjusted our analysis for smoking status, dietary intake, and physical activity. However, adding these lifestyle factors to our models did not meaningfully change our risk estimates. This analysis was based on data from a multicenter population‐based prospective cohort study. Although we do not know whether patients with NMIBC included in the current analysis differed from invited eligible patients with NMIBC regarding body weight, they were comparable with respect to age, sex, and tumor characteristics.

## 5 CONCLUSION

In conclusion, general and abdominal obesity were not associated with an increased risk of recurrence among patients with NMIBC but tended to increase risk of progression. As current evidence is inconsistent, further studies with obesity measures, preferably also including CT‐based visceral and subcutaneous fat, and with sufficient sample size to stratify by tumor stage and type of treatment are needed to understand whether and how obesity after diagnosis could influence risk of recurrence and progression.

## AUTHOR CONTRIBUTIONS

**Moniek van Zutphen:** Conceptualization (equal); data curation (equal); formal analysis (lead); methodology (equal); visualization (lead); writing – original draft (lead); writing – review and editing (lead). **Ivy Beeren:** Data curation (equal); investigation (lead); writing – review and editing (supporting). **Katja KH Aben:** Resources (equal); writing – review and editing (supporting). **Antoine G van der Heijden:** Resources (equal); writing – review and editing (equal). **J. Alfred Witjes:** Resources (equal); writing – review and editing (supporting). **Lambertus A.L.M. Kiemeney:** Conceptualization (equal); writing – review and editing (supporting). **Alina Vrieling:** Conceptualization (equal); funding acquisition (lead); methodology (equal); project administration (lead); supervision (lead); writing – review and editing (equal).

## FUNDING INFORMATION

This work was supported by Alpe d'HuZes/Dutch Cancer Society (KUN 2013–5926), Dutch Cancer Society (2017–2/11179), and World Cancer Research Fund International and Wereld Kanker Onderzoek Fonds (IIG_2019_1957). The funding sources were not involved in study design, in the collection, analysis and interpretation of data, in the writing of the manuscript, and the decision to submit the article for publication.

## CONFLICT OF INTEREST STATEMENT

The authors declare no conflict of interest.

## ETHICS STATEMENT

This study was performed in line with the principles of the Declaration of Helsinki. Ethical approval was provided by the Committee for Human Research region Arnhem‐Nijmegen (CMO 2013–494). Informed consent was obtained from all individual participants included in the study.

## Supporting information


Supplementary Table 1.

## Data Availability

Data availability: Data described in the manuscript, code book, and analytic code will be made available upon request pending application to and approval from the corresponding author A.V.
