# Supplementary material for: Body mass index and waist circumference in relation to risk of recurrence and progression after non‐muscle invasive bladder cancer
Source: Cancer Med. 2023 Oct 6;12(20):20459–69. doi: 10.1002/cam4.6620 (PMC10652337; doi:10.1002/cam4.6620)
Supplement: Supplementary file 1 — Supplementary Table 1. [file CAM4-12-20459-s001.docx]

Supplementary file belonging to:

**Body mass index and waist circumference in relation to risk of recurrence and progression after non-muscle invasive bladder cancer**

M. van Zutphen, I. Beeren, K.K.H. Aben, A.G. van der Heijden, J.A. Witjes, L.A.L.M. Kiemeney, A. Vrieling.

Alina Vrieling, Department for Health Evidence, Radboud university medical center, Nijmegen, The

Netherlands; Alina.Vrieling@radboudumc.nl

Supplementary Table 1

Hazard ratios (HR) for the association of obesity with risk of multiple bladder cancer recurrences

**Supplementary Table 1.** Hazard ratios (HR) for the association of obesity with risk of multiple bladder cancer recurrences

|  | N | Events / person-years | HR (95%CI) |
| --- | --- | --- | --- |
| Measure |  |  |  |
| **BMI 2y prediagnosis, kg/m^2^** |  |  |  |
| 18.5 to <25.0 | 298 | 201/1193 | 1.00 (ref) |
| 25.0 to <30.0 | 460 | 253/1828 | 0.84 (0.68, 1.06) |
| ≥30 | 188 | 98/752 | 0.80 (0.60, 1.07) |
| P‑trend |  |  | 0.12 |
| Per 5 kg/m^2^ | 946 | 552/3773 | 0.90 (0.80, 1.02) |
| **BMI 3mo postdiagnosis, kg/m^2^** |  |  |  |
| 18.5 to <25.0 | 332 | 198/1306 | 1.00 (ref) |
| 25.0 to <30.0 | 500 | 283/2003 | 0.95 (0.77, 1.19) |
| ≥30 | 197 | 110/792 | 0.94 (0.71, 1.24) |
| P‑trend |  |  | 0.65 |
| Per 5 kg/m^2^ | 1029 | 591/4102 | 0.98 (0.87, 1.11) |
| **Waist circumference 3mo postdiagnosis, cm** |  |  |  |
| <94 M and <80 F | 204 | 128/808 | 1.00 (ref) |
| 94 to <102 M and 80 to <88 F | 257 | 155/1024 | 0.98 (0.74, 1.30) |
| ≥102 M and ≥88 F | 445 | 238/1786 | 0.86 (0.66, 1.11) |
| P‑trend |  |  | 0.19 |
| Per 10 cm | 906 | 521/3618 | 0.97 (0.89, 1.06) |
| **Waist-to-hip ratio**  **3mo postdiagnosis** |  |  |  |
| Sex-specific tertile 1^a^ | 296 | 178/1171 | 1.00 (ref) |
| Sex-specific tertile 2^b^ | 306 | 181/1215 | 0.99 (0.77, 1.26) |
| Sex-specific tertile 3^c^ | 303 | 162/1227 | 0.87 (0.68, 1.12) |
| P‑trend |  |  | 0.24 |
| Per 0.1 unit | 905 | 521/3613 | 0.94 (0.81, 1.10) |
| **Pre-to-post diagnosis weight change** |  |  |  |
| Weight loss (≥5%) | 117 | 53/469 | 0.75 (0.54, 1.04) |
| Weight stable (-5% to 5%) | 637 | 394/2524 | 1.00 (ref) |
| Weight gain (≥5%) | 85 | 39/354 | 0.77 (0.53, 1.13) |
| P-trend |  |  | 0.67 |
| Per 5% weight gain | 839 | 486/3347 | 1.08 (0.98, 1.20) |

Adjusted for age at diagnosis and sex.

^a^ Cut-offs: <0.967 M (male) and <0.861 F (female).

^b^ Cut-offs: 0.967 to 1.019 M and 0.861 to 0.926 F.

^c^ Cut-offs: >1.019 M and >0.926 F.
